# EpCAM nuclear localization identifies aggressive Thyroid Cancer and is a marker for poor prognosis

**DOI:** 10.1186/1471-2407-10-331

**Published:** 2010-06-25

**Authors:** Ranju Ralhan, Jun Cao, Terence Lim, Christina MacMillan, Jeremy L Freeman, Paul G Walfish

**Affiliations:** 1 Department of Otolaryngology, Head & Neck Surgery Program, Joseph and Mildred Sonshine Family Centre for Head and Neck Diseases, Mount Sinai Hospital, Joseph and Wolf Lebovic Health Complex, 600 University Avenue, Toronto, ON, M5G 1X5, Canada; 2 Alex and Simona Shnaider Research Laboratory in Molecular Oncology, Mount Sinai Hospital, Joseph and Wolf Lebovic Health Complex, 600 University Avenue, Toronto, ON, M5G 1X5, Canada; 3 Department of Pathology and Laboratory Medicine, Mount Sinai Hospital, Joseph and Wolf Lebovic Health Complex, 600 University Avenue, Toronto, ON, M5G 1X5, Canada; 4 Department of Medicine, Endocrine Division, Mount Sinai Hospital, Joseph and Wolf Lebovic Health Complex, 600 University Avenue, Toronto, Ontario, M5G 1X5, Canada; 5 Department of Otolaryngology, Head and Neck Surgery, University of Toronto, 190 Elizabeth Street, Fraser Elliot Building, Toronto, Ontario, M5G 2N2, Canada

## Abstract

**Background:**

Proteolytic cleavage of the extracellular domain (EpEx) of Epithelial cell adhesion molecule (EpCAM) and nuclear signaling by its intracellular oncogenic domain Ep-ICD has recently been implicated in increased proliferation of cancer cells. The clinical significance of Ep-ICD in human tumors remains an enigma.

**Methods:**

EpEx, Ep-ICD and β-catenin immunohistochemistry using specific antibodies was conducted on 58 archived thyroid cancer (TC) tissue blocks from 34 patients and correlated with survival analysis of these patients for up to 17 years.

**Results:**

The anaplastic (ATC) and aggressive thyroid cancers showed loss of EpEx and increased nuclear and cytoplasmic accumulation of Ep-ICD. In contrast, the low grade papillary thyroid cancers (PTC) showed membranous EpEx and no detectable nuclear Ep-ICD. The ATC also showed concomitant nuclear expression of Ep-ICD and β-catenin. Kaplan-Meier Survival analysis revealed reduced overall survival (OS) for TC patients showing nuclear Ep-ICD expression or loss of membranous EpEx (p < 0.0004), median OS = 5 months as compared to 198 months for patients who did not show nuclear Ep-ICD or demonstrated only membranous EpE.

**Conclusion:**

We report reciprocal loss of membrane EpEx but increased nuclear and cytoplasmic accumulation of Ep-ICD in aggressive TC; nuclear Ep-ICD correlated with poor OS of TC patients. Thus nuclear Ep-ICD localization may serve as a useful biomarker for aggressive TC and may represent a novel diagnostic, prognostic and therapeutic target for aggressive TC.

## Background

Epithelial cell adhesion molecule (EpCAM) is a 40kDa transmembrane glycoprotein frequently overexpressed in human malignancies, normal stem and progenitor cells, cancer-initiating cells in breast, colon, pancreas and prostate carcinomas and albeit at lower levels in normal epithelia [1-11]. There is a large database on EpCAM staining for many cancers and normal tissues. However, all these studies used antibodies directed against the extracellular domain (EpEx) of EpCAM that detected the EpCAM precursor or cell-bound EpEx, or both [3]. EpCAM serves important roles in cell adhesion, proliferation, differentiation, migration, cell cycle regulation and is implicated in cancer and stem cell signaling [12].

Regulated intramembrane proteolysis has recently been shown to act as the mitogenic signal transducer of EpCAM *in vitro *and *in vivo *[13]. The cleavage and shedding of EpCAM ectodomain, EpEx, by proteases- TACE and Presenilin-2, has been shown to release its intracellular domain (Ep-ICD) that translocates to the nucleus. The association of Ep-ICD with FHL2 and Wnt pathway components - β-catenin and Lef-1 forms a nuclear complex that binds DNA at Lef-1 consensus sites and induces gene transcription, leading to increased cell proliferation and has been shown to be oncogenic in immunodeficient mice [13]. In view of the novel role of EpCAM as an oncogenic signal transducer and cancer stem cell marker [12,14-16], it is important to establish the clinical significance of nuclear Ep-ICD in human cancers. Nuclear Ep-ICD was recently reported in a preliminary study in human colon cancer, but not in the normal colonic epithelium [13]. In view of the tremendous heterogeneity in solid tumors, the clinical significance of nuclear Ep-ICD in other human cancers needs to be established.

Thyroid cancer (TC) represents 90% of all endocrine malignancies with an estimated annual incidence of 122,800 cases worldwide and approximately 33,000 newly diagnosed cases in USA [17]. Anaplastic thyroid cancer (ATC) is a rare but very aggressive form of this malignancy, accounting for less than 2% of all TC. ATC commonly presents as a rapidly increasing neck mass that spreads locally, compresses the adjacent structures, with a tendency to disseminate to regional lymph nodes and distant sites [18,19]. Most well differentiated TC have an excellent prognosis, with relative 5-year survival rates above 95%, despite their tendency for early metastasis. However, the less-differentiated thyroid tumors - ATC and other aggressive metastatic TC can be fatal with median survival time ranging from 4 months to 5 years [19]. This variation in clinical outcomes remains a major challenge that may be attributed to the differences in genetic damage acquired by the aggressive and non-aggressive TC during their malignant evolution [20-22].

β-Catenin plays important roles in cell adhesion and signal transduction [23]. β-Catenin associates with E-cadherin and α-catenin linking the adherens junctions and cytoskeleton, besides acting as a mediator of transcription through DNA-binding proteins, such as TCF/LEF family members in the nucleus [24]. Loss of membrane-associated β-catenin and a relative increase in cytosolic or nuclear expression, has been reported in anaplastic and poorly differentiated TCs and in thyroid papillary microcarcinoma [25-27]. β-catenin -RET kinase pathway has been shown to be a critical contributor to the development and metastasis of human thyroid carcinoma [28]. Immunohistochemical analysis of EpEx (using the monoclonal antibody 17-1A directed against extracellular domain of EpCAM) showed membrane staining in differentiated TC and poorly differentiated TC. In contrast the ATC completely lacked EpEx expression [29]. In view of the aggressive nature of ATC these findings are puzzling. To address this challenge we sought to investigate EpEx and Ep-ICD protein expression in human primary TC by immunohistochemistry (IHC) using antibodies directed against Ep-Ex and Ep-ICD domains of EpCAM.

Further, concurrent staining for nuclear β-catenin was carried out to establish its correlation with the oncogenic Ep-ICD signaling in TC. To our knowledge our study is the first report demonstrating the clinical relevance of nuclear Ep-ICD and its putative utility as an adverse prognosticator in human TC.

## Methods

### Patients and tissue specimens

The study was approved by Mount Sinai Hospital Research Ethics Board, Toronto, Canada. Fifty eight TC formalin fixed paraffin embedded (FFPE) tissue blocks obtained from 34 patients were retrieved from the archives of the Department of Pathology, Mount Sinai Hospital, Toronto, Canada. Each case was reviewed by the pathologist prior to further experiments. The TC blocks analyzed included- 19 ATC, 4 insular carcinomas (poorly differentiated), 1 poorly differentiated papillary thyroid cancer (PDPTC), 1 poorly differentiated follicular thyroid cancer (PDFTC), 24 PTC, 3 FTC, 4 squamous cell carcinoma (SCC) and 2 normal thyroid tissues. The patient follow up data were retrieved from the clinical data base of one of us (PGW) to correlate the protein expression in tumors with clinical outcome to evaluate the prognostic relevance of these proteins. The patients were followed up for a minimum period of 15 months and a maximum period of 17 years.

### Antibodies

Anti human-EpCAM mouse monoclonal antibody MOC-31(AbD Serotec, Oxford, UK) recognizes an extracellular component (EGF1 domain- aa 27-59) in the amino-terminal region of EpCAM [30]. Intracellular domain of EpCAM, α-EpICD antibody 1144 [Epitomics Inc. (Burlingame, CA)] recognizes the cytoplasmic domain of human EpCAM. β-catenin antibody was raised against aa 571-781 of β-catenin (Cat.# 610154, B D Sciences, San Jose, CA).

### Immunohistochemistry for EpEx and Ep-ICD expression in thyroid cancers

Serial TC tissue sections (4 μm thickness) were deparaffinized, hydrated in xylene and graded alcohol series. The slides were treated with 0.3% H2O2 at room temperature for 30 minutes to block the endogenous peroxidase activity. After blocking the non-specific binding with normal horse or goat serum, the sections were incubated with anti human antibodies -EpEx mouse monoclonal antibody MOC-31 (dilution 1:200), or α- EpICD rabbit monoclonal antibody 1144 (dilution 1:200), or mouse monoclonal β-catenin antibody (dilution 1:200) for 30 minutes and biotinylated secondary antibody (horse anti-mouse or goat anti-rabbit) for 30 minutes. The sections were finally incubated with VECTASTAIN Elite ABC Reagent (Vector laboratories, Burlington, Ontario, Canada) and diaminobenzedine was used as the chromogen.

### Evaluation of immunohistochemical staining

Immunopositive staining was evaluated in five areas of the tissue sections as described [31]. Sections were scored as positive if epithelial cells showed immunopositivity in the plasma membrane, cytoplasm, and/or nucleus when observed by two evaluators who were blinded to the clinical outcome. These sections were scored as follows: 0, < 10% cells; 1, 10-30% cells; 2, 30-50% cells; 3, 50-70% cells; and 4, >70% cells showed immunoreactivity. Sections were also scored semi-quantitatively on the basis of intensity as follows: 0, none; 1, mild; 2, moderate; and 3, intense. Finally, a total score (ranging from 0 to 7) was obtained by adding the scores of percentage positivity and intensity for each of the thyroid cancer and normal thyroid tissue sections. The immunohistochemical data were subjected to statistical analysis as described previously [31]. The immunohistochemical scoring data were verified using the Visiopharm Integrator System (Visiopharm, Horsholm, Denmark). Only the nuclear staining was quantitated, as the software did not permit simultaneous quantitation of membranous, cytoplasmic and nuclear staining based on differences in intensity of positive brown staining.

### Statistical analysis

The immunohistochemical data were subjected to statistical analysis using SPSS 10.0 software (SPSS Inc., Chicago, IL). Box plots were used to determine the distribution of total score of membranous EpEx, nuclear Ep-ICD and nuclear or cytoplasmic β-catenin expression in normal thyroid tissues and thyroid cancers. A cut-off = or > 2 was defined as positive criterion for nuclear β-catenin immunopositivity for statistical examination. For membranous β-catenin, score of 6 was defined as loss of expression. The correlation between expression of EpEx, Ep-ICD and/or β-catenin staining with overall patient survival was evaluated using life tables constructed from survival data with Kaplan- Meier plots.

## Results

### Immunohistochemical Analysis of EpEx and Ep-ICD expression in Thyroid Cancer

**Figure 1:**
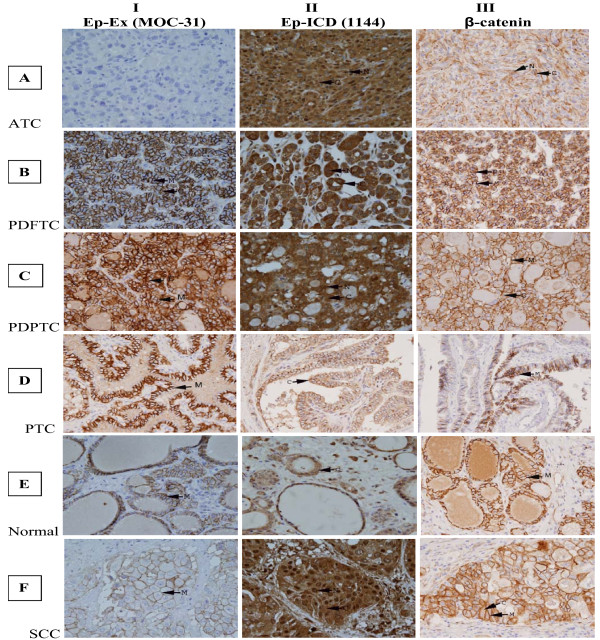
**Immunohistochemical analysis of EpEx, Ep-ICD and β catenin in thyroid cancer**. The ATC did not show detectable membranous EpEx staining (IA); all the other subtypes of TC analyzed and normal thyroid tissues showed varying levels of plasma membranous EpEx staining (IB-IF). Nuclear Ep-ICD staining was observed in undifferentiated and poorly differentiated TC (IIA-IIC, and IIF), but not in well differentiated TC and the adjacent normal thyroid tissue (IID, IIE). Correlated with nuclear Ep-ICD staining, nuclear or cytoplasmic β catenin staining was observed in aggressive TC (IIIA-IIIC, and IIIF), while membranous staining was observed in the less aggressive TC and the adjacent normal thyroid tissues (IIID, IIIE). Panels A, B and F- I and II, C and F III- original magnification × 40; panels A III, B III, C, D, E - I and II, D and E III- original magnification × 20. The abbreviations M, C, and N denote membranous, cytoplasmic and nuclear localization of the proteins.

To determine the clinical significance of Ep-Ex and Ep-ICD in TC, their expressions were analyzed in archived tissues by immunohistochemistry using domain specific antibodies against EpEx (MOC-31) and Ep-ICD (1144) respectively. The immunostaining scores of EpEx, Ep-ICD, and beta-catenin in individual tissue sections are given in Table 1. No plasma membrane EpEx immunoreactivity was observed in ATC (Fig. 1, panel IA); intense nuclear and cytoplasmic Ep-ICD immunostaining was observed in ATC (Fig. 1, panel IIA). β-catenin immunostaining was carried out in serial sections to determine if there was any correlation between cytoplasmic/nuclear Ep-ICD and nuclear/cytoplasmic β-catenin. Our study showed concurrent cytoplasmic and nuclear β-catenin immunostaining in ATC (Fig. 1, panel IIIA). In comparison, a subset of the poorly differentiated follicular TC (PDFTC) showed moderate Ep-ICD cytoplasmic and nuclear staining (Fig. 1panel IIB); predominant membrane and mild cytoplasmic staining was observed for β-catenin (Fig. 1, panel IIIB). The PDPTC showed EpEx membrane staining (Fig. 1, panel IC); Ep-ICD staining was detected in some tumor nuclei and less intense cytoplasmic immunoreactivity was also observed in these tumors (Fig. 1, panel IIC); in comparison only membrane and mild cytoplasmic β-catenin staining was observed (Fig. 1, panel IIIC). The well differentiated PTC (WDPTC) showed intense EpEx membrane staining (Fig. 1, panel ID) but no Ep-ICD nuclear staining and only mild cytoplasmic immunostaining was observed in these tumors (Fig. 1, panel IID) and only intense membrane staining was observed for β-catenin (Fig. 1, panel IIID). In comparison the normal (non-malignant) thyroid tissues showed basal membrane EpEx immunoreactivity (Fig. 1, panel IE), faint or absent cytoplasmic/nuclear Ep-ICD staining (Fig. 1, panel IIE) and basal membrane immunostaining for β-catenin (Fig. 1, panel IIIE). The squamous cell carcinoma variant showed faint EpEx membrane immunoreactivity (Fig. 1, panel IF); intense cytoplasmic and nuclear Ep-ICD staining (Fig. 1, panel IIF); and membranous and cytoplasmic immunoreactivity for β-catenin (Fig. 1, panel IIIF). The symbols M, C, N refer to membrane, cytoplasmic and nuclear localization of the protein.

**Table 1:** IHC Scoring of EpEx, Ep-ICD and β-catenin in Thyroid cancers

S No.	Subtype	EpEx	EpEx	EpEx	Ep-ICD	Ep-ICD	EP-ICD	β-catenin	β-catenin	β-catenin
		**M**	**C**	**N**	**M**	**C**	**N**	**M**	**C**	**N**

1	Normal	7	6	0	0	5	0	7	5	0
2	Normal	7	6	0	0	6	0	6	4	0
3	PTC	7	4	0	3	5	0	5	4	0
4	PTC	7	5	0	5	5	0	5	0	0
5	PTC	7	4	0	0	5	0	6	5	0
6	PTC	7	5	0	0	6	0	6	6	0
7	PTC	7	5	0	0	5	3	7	3	0
8	PTC	6	3	0	3	6	0	6	5	0
9	PTC	7	6	0	0	4	0	4	3	0
10	PTC	7	6	0	4	5	0	7	0	0
11	PTC	7	5	0	0	7	0	7	5	0
12	PTC	7	5	0	0	6	0	7	5	0
13	PTC	7	6	0	0	5	0	7	5	0
14	PTC	7	5	0	2	5	0	5	0	0
15	PTC	7	4	0	0	5	0	5	2	0
16	PTC	6	5	0	2	5	0	5	0	0
17	PTC	6	4	0	0	4	0	5	0	0
18	PTC	7	5	0	3	6	0	7	3	0
19	PTC	7	6	0	4	5	0	6	4	0
20	PTC	7	5	0	0	6	0	7	6	0
21	PTC	7	6	0	0	6	0	7	5	0
22	PTC	7	6	0	3	6	0	7	6	0
23	PTC	7	4	0	0	6	0	4	4	0
24	PTC	6	4	0	0	5	0	3	2	0
25	PTC	6	4	0	0	6	0	6	4	0
26	PTC	7	5	0	0	5	0	7	6	0
27	FTC	6	5	0	0	5	0	7	2	0
28	FTC	2	3	0	7	3	0	7	3	0
29	FTC	6	5	0	0	4	0	7	6	0
30	PDPTC	7	5	0	0	6	2	6	5	0
31	PDFTC	7	5	0	2	5	2	6	5	0
32	SCC	2	2	0	0	5	4	5	4	0
33	SCC	3	2	0	0	5	0	5	4	0
34	SCC	4	3	0	0	5	3	5	4	0
35	SCC	5	3	0	0	5	4	5	4	0
36	Insular	0	0	0	3	4	0	0	0	0
37	Insular	0	0	0	5	4	0	0	0	0
38	Insular	7	5	0	5	5	2	6	4	0
39	Insular	7	4	0	0	5	0	7	6	0
40	ATC	0	0	0	0	5	6	2	6	0
41	ATC	0	0	0	0	5	6	0	2	3
42	ATC	0	0	0	0	3	7	0	2	0
43	ATC	0	0	0	0	2	2	0	0	0
44	ATC	0	0	0	0	2	7	0	2	0
45	ATC	0	0	0	0	3	3	5	2	0
46	ATC	0	0	0	0	0	2	2	4	0
47	ATC	0	0	0	0	3	5	3	4	0
48	ATC	0	0	0	0	2	2	0	0	0
49	ATC	0	0	0	0	5	4	3	5	0
50	ATC	0	0	0	0	5	5	0	5	5
51	ATC	0	0	0	0	5	6	0	4	5
52	ATC	0	0	0	0	5	5	0	2	2
53	ATC	0	0	0	0	4	5	0	4	4
54	ATC	0	0	0	0	4	4	0	3	3
55	ATC	0	0	0	0	4	4	3	4	2
56	ATC	0	0	0	0	5	3	0	2	2
57	ATC	0	0	0	0	4	4	5	6	4
58	ATC	7	5	0	6	5	2	0	0	0

**Figure 2:**
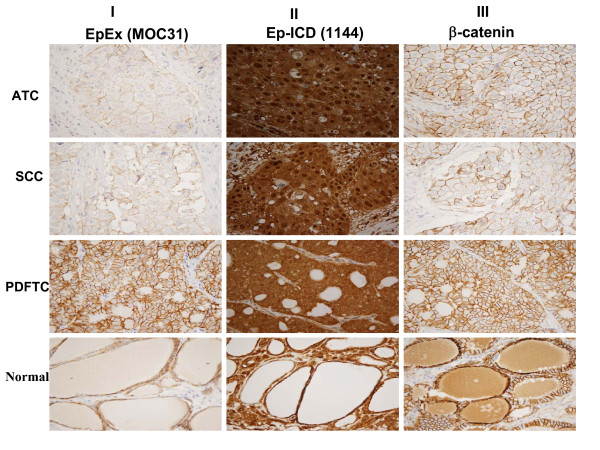
**Differential immunohistochemical expression of EpEx, Ep-ICD and β-catenin proteins in different regions of an ATC tumor with varying degrees of aggressive tumor pathology**. No membranous EpEx staining was observed in the ATC tissue section (IA), faint membranous EpEx staining in tissue section showing squamous cell carcinoma (IB), membranous EpEx staining in both poorly differentiated TC section and the adjacent normal thyroid tissue section (IC, ID). Nuclear and cytoplasmic Ep-ICD staining in undifferentiated and poorly differentiated TC tissue sections (IIA-IIC), membranous and cytoplasmic staining in the adjacent thyroid normal tissue (IID). Nuclear and cytoplasmic β catenin staining in ATC section (IIIA), membranous β catenin staining in different regions of this tumor with varying degrees of aggressive tumor pathology (IIIB-IIID). A-D, original magnification × 10.

Illustrated in Fig. 2 is a pattern of differential expression of these proteins in different regions of an ATC tumor with varying degrees of aggressive tumor pathology. The panel AI depicts an ATC section showing no EpEx membrane staining, while the panel AII shows intense nuclear and cytoplasmic localization of Ep-ICD in the serial ATC section and panel AIII shows membrane, cytoplasmic and nuclear β-catenin expression. Another tissue block from the same patient showed SCC - Panel BI shows focal faint membrane EpCAM expression, while Panel BII shows intense nuclear and cytoplasmic Ep-ICD staining and Panel BIII shows nuclear and membranous β-catenin expression. In comparison another tissue block from the same patient showing features of PDFTC as demonstrated by only membranous EpEx (Panel CI) and only cytoplasmic Ep-ICD was observed (Panel CII) and membranous and cytoplasmic β-catenin was observed (Panel CIII). The adjacent normal thyroid tissue from this patient showed membranous EpEx staining (Panel DI) without Ep-ICD nuclear staining (Panel DII) and only membranous staining for β-catenin (Panel DIII). These different staining patterns observed in the same patient support the influence of different subcellular expression patterns which correlate with varying degrees of aggressive pathology in different subsets of TC.

### Box-Plot analysis

**Figure 3:**
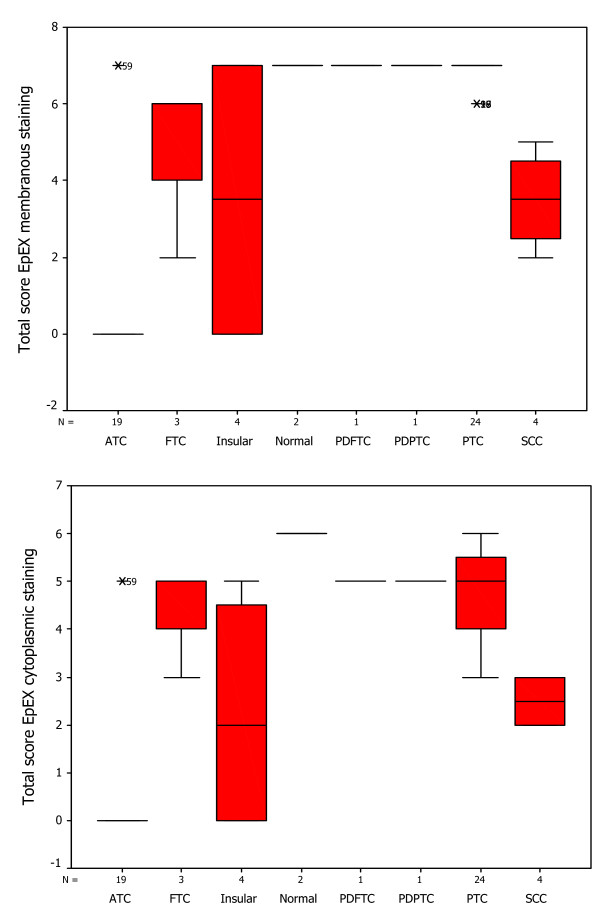
**Box-Plot analysis of EpEx expression in thyroid cancers**. Box plots showing distribution of total immunostaining scores determined by immunohistochemistry in paraffin-embedded sections of normal thyroid tissues and different types of thyroid cancers. The vertical axis gives the total immunostaining score, obtained as described in the Methods section. Upper panel depicts the membranous EpEx localization in normal tissues and PTC, no detectable expression in ATC and varying reduced expressions in Insular, FTC and SCC (with a median score of 3, bold horizontal line). The lower panel depicts cytoplasmic EpEx localization in normal tissues, PTC, PDPTC, PDFTC, insular and FTC, no detectable expression in ATC and varying reduced expression in SCC. No detectable nuclear EpEx staining was observed in normal tissues, or in any of the subtypes of TC, hence this box plot was not included in the figure.

**Figure 4:**
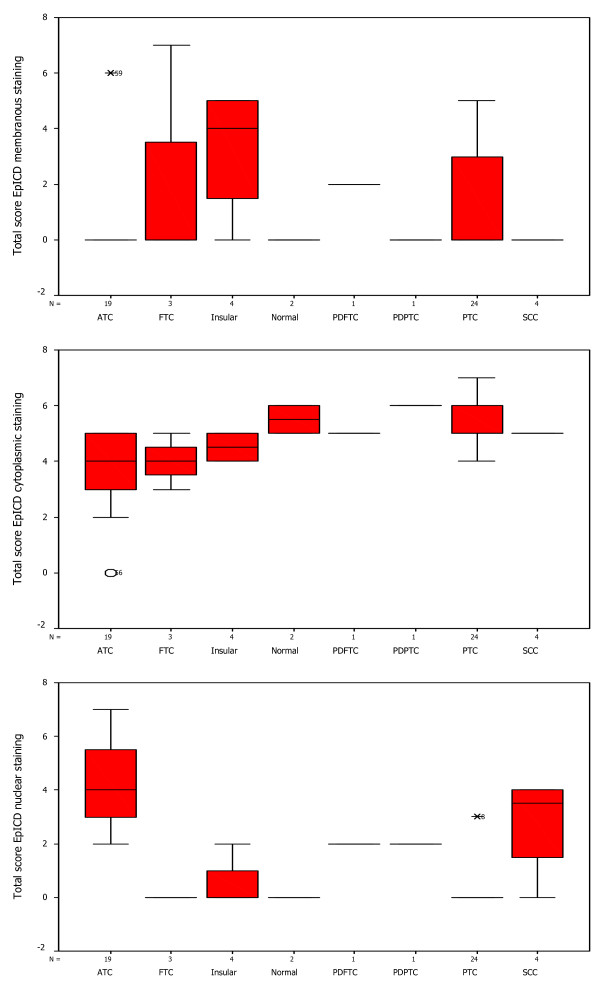
**Box-Plot analysis of Ep-ICD expression in thyroid cancers**. The upper panel shows box plots for membranous Ep-ICD localization in normal tissues, some PTC, insular, PDFTC and PDPTC, but no membranous staining in ATC, FTC and SCC. The middle panel depicts cytoplasmic Ep-ICD localization in normal tissues, PTC, ATC, FTC, insular and SCC, PDPTC and PDFTC. The lower panel depicts nuclear Ep-ICD localization in ATC and varying expression in SCC, (with a median score of 3, bold horizontal line, range 0-4, as shown by vertical bars), as compared to PTC, insular, FTC, PDPTC, PDFTC and normal thyroid tissues with a median score of 0.

**Figure 5:**
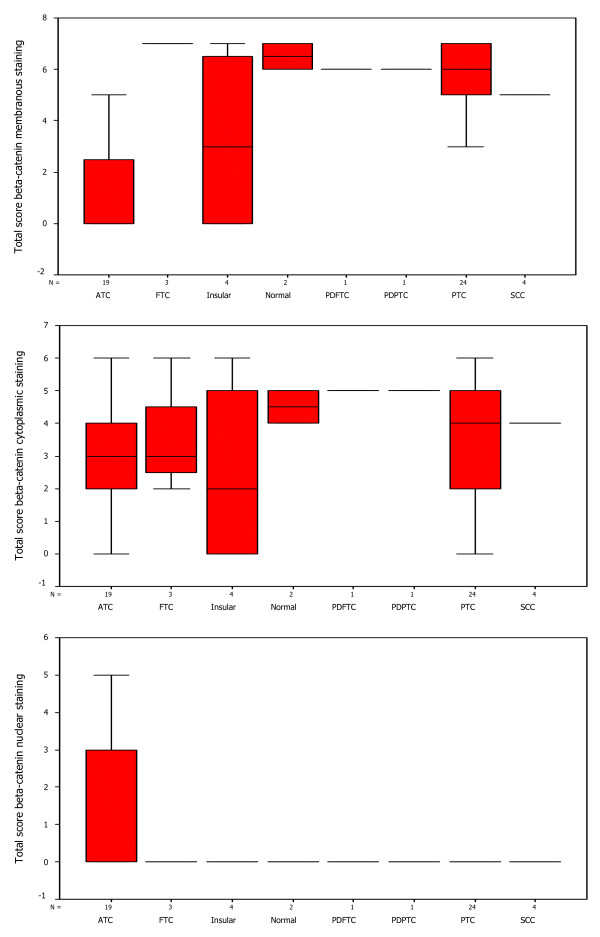
**Box-Plot analysis of β-catenin expression in thyroid cancers**. The upper panel shows box plots for membranous β-catenin staining in ATC only. The middle panel shows cytoplasmic β-catenin in all the subtypes of TC analyzed. The lower panel shows nuclear β-catenin in normal tissues and all the subtypes of TC analyzed except most of the ATCs.

**Figure 6:**
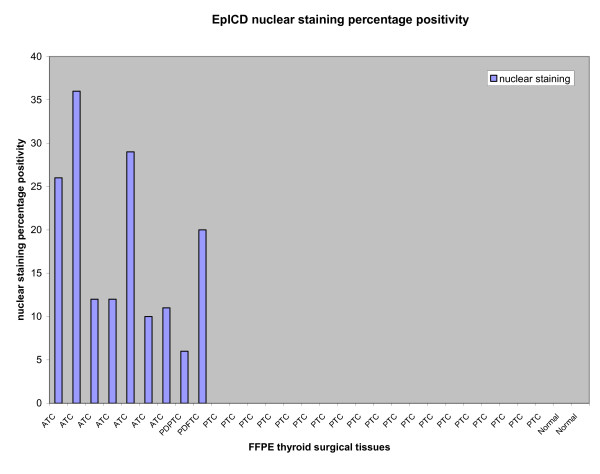
**Ep-ICD nuclear staining in different subtypes of thyroid cancers using the Visiopharm Integrator System**. Representative histogram showing nuclear Ep-ICD expression in ATC, PDPTC and PDFTC analyzed, while no nuclear Ep-ICD accumulation was observed in the PTCs or the normal thyroid tissues analyzed.

The distribution of total immunostaining scores of EpEx, Ep-ICD and β-catenin, determined in sections of normal thyroid tissues and different subtypes of TC are shown in Figures 3, 4, 5. The nuclear Ep-ICD staining was quantified using the Visiopharm Integrator System; the histogram showing percentage nuclear Ep-ICD positivity in all the different subtypes of TC is given in Fig. 6. The ATC, insular, PDPTC and PDFTC analyzed showed nuclear Ep-ICD expression, the total nuclear Ep-ICD positive area ranged from 12- 40%, while the PTC did not show nuclear expression. Overall, analysis of β-catenin expression in different subtypes of TC showed predominantly cytoplasmic expression though some nuclear expression was also observed in ATC. In comparison, membrane localization of β-catenin was observed in most of the PDFTC, PDPTC and WDPTC (except for a small subset) as well as in the non-malignant thyroid tissues.

### Correlation of Ep-ICD and EpEx expression with disease outcome

**Figure 7:**
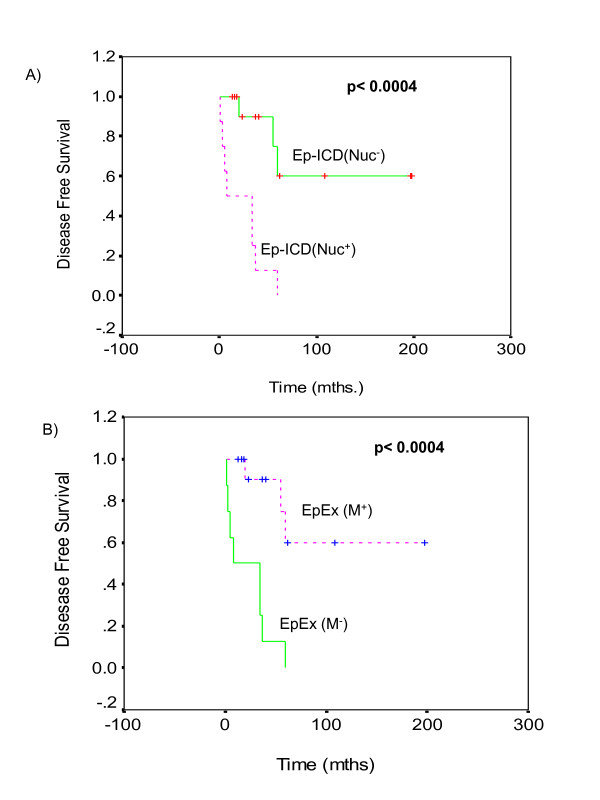
**Kaplan-Meier estimation of cumulative proportion of overall survival: (A) Nuclear Ep-ICD accumulation in thyroid cancers correlated significantly with reduced overall survival (OS) of these patients (p < 0.0004)**. The median OS was 5 months in the group of patients showing nuclear Ep-ICD as compared to 198 months for patients who did not have detectable nuclear Ep-ICD. (B) Loss of membranous EpEx expression was also associated with reduced overall survival (OS) for patients. The patients showing loss of membranous EpEx had shorter OS (median = 5months) than those showing membranous expression (median = 198 months, p < 0.0004).

Kaplan-Meier Survival analysis of the 34 TC patients in this study revealed reduced overall survival (OS) for patients showing nuclear Ep-ICD expression (p < 0.0004, Fig. 7A). The median OS was 5 months in patients showing nuclear Ep-ICD as compared to 198 months for patients who did not. Further, patients showing loss of membranous EpEx also had shorter OS (median = 5 months) than those showing membranous expression (median = 198 months, p < 0.0004, Fig. 7B).

## Discussion

The key findings of our study are: (i) The ATC showed loss of membrane EpEx, but increased Ep-ICD accumulation in cytoplasm and nucleus of tumor cells. These tumors also showed concomitant nuclear β catenin expression. Our observations strongly suggest that Ep-ICD may be acting as an oncogenic signal transducer in these tumors by activation of Wnt pathway components including β catenin to promote rapid growth of these tumors and a poor prognosis; (ii) EpEx membrane overexpression was observed in both well differentiated- follicular and papillary TC, where as only a small subset of poorly differentiated- follicular and papillary TC showed nuclear Ep-ICD localization. (iii) TC patients showing nuclear Ep-ICD expression or loss of membranous EpEx showed reduced OS (p < 0.0004, median OS = 5 months as compared to 198 months for patients who did not show nuclear Ep-ICD or showed membranous EpEx). Interestingly, our study is the first report using an antibody specific for the cytoplasmic domain of Ep-ICD that demonstrates its cytoplasmic and nuclear accumulation in ATC. The regulated intramembrane proteolysis (RIP) of EpCAM has recently been proposed to produce Ep-ICD that has been shown to transduce EpCAM signaling in cancer cells and activate Wnt proteins-resulting in increased nuclear accumulation of β-catenin and the target genes - c-myc and cyclinD1[13].

Demonstration of concomitant nuclear expression of Ep-ICD and β-catenin in ATC supports the hypothesis that activation of Ep-ICD signaling also increased the Wnt pathway component activation and could account for the increased oncogenic signaling in ATC. β-catenin plays an important role as a signaling factor involved in canonical Wnt pathway [32]. Nuclear localization of β-catenin is involved in precancerous change in oral leukoplakia [33], and is known to associate with malignant transformation of human cancers including colorectal, gastric and esophageal tumors [34-37]. The activation of canonical Wnt signaling pathway results in nuclear translocation of β-catenin [38]. Hence nuclear β-catenin is a marker for active cell proliferation. In contrast to membranous and cytoplasmic expression, nuclear localization of β-catenin is implicated in tumor progression. The nuclear β-catenin expression in ATC also supports the aggressive nature of such tumors.

Most remarkably, the survival analysis data showed a correlation between nuclear Ep-ICD accumulation and reduced OS of TC patients (p < 0.0004). Further, loss of membranous EpEx also correlated with reduced OS of TC patients (p < 0.0004), OS (median = 5 months) as compared to those TC patients who did not show nuclear accumulation of these proteins (median = 198 months). However, to our knowledge this is the first report underscoring the clinical significance of nuclear Ep-ICD as an adverse prognosticator for aggressive TC. Although the numbers of ATC analyzed in our study is small, our novel observations on the clinical correlation of Ep-ICD nuclear localization as an oncogenic signal of TC appears to be a quite striking and consistent finding to date.

Our observations of loss of EpEx expression on the plasma membrane of ATC is in accord with an earlier report on EpCAM expression in TC by Ensinger et al., [29]. Our results using MOC-31, an antibody that recognizes the extracellular domain of Ep-CAM, also confirm the surprising loss of EpCAM expression from the plasma membrane in ATC. There are numerous reports in the literature on the cell membrane expression of EpCAM in TC and other human cancers [8-10,29,30,39-43], leading to the suggestion that it could be an ideal candidate for application as a cancer marker and a therapeutic target. However, the loss of membrane EpEx and the increased nuclear and cytoplasmic localization of Ep-ICD in ATC and other aggressive TC suggest that another therapeutic approach targeting Ep-ICD is likely to be more effective in the treatment of such aggressive TC.

## Conclusions

In conclusion, we demonstrate loss of membranous EpEx and increased nuclear and cytoplasmic accumulation of Ep-ICD in a selected subset of aggressive TC (ATC and some PDPTC and PDFTC). A concomitant increase in nuclear β-catenin in these aggressive carcinomas suggests a pathogenic role of Wnt signaling in these malignancies. Further, either loss of membranous EpEx, or nuclear accumulation of Ep-ICD correlated with poor OS of such TC patients. We propose that nuclear Ep-ICD may serve as a putative biomarker for aggressive TC and functions as a potential target for novel diagnostic, prognostic and therapeutic strategies in the management of aggressive TC.

## Abbreviations

EpCAM: Epithelial cell adhesion molecule; EpEx: extracellular domain; Ep-ICD: intracellular domain of EpCAM; TC: thyroid cancer; PTC: papillary thyroid cancer; ATC: anaplastic thyroid cancers; IHC: immunohistochemistry; PDPTC: poorly differentiated papillary thyroid cancer; PDFTC: poorly differentiated follicular thyroid cancer; SCC: squamous cell carcinoma; WDPTC: well differentiated PTC; OS: overall survival.

## Competing interests

The authors declare that they have no competing interests.

## Authors' contributions

RR conceptualized, designed and conducted the study, carried out the interpretation and data analysis, and wrote the manuscript. JC carried out the experimental work, data analysis, photomicrography and wrote the manuscript. TL conducted the chart reviews and provided clinical data for all the patients. CM performed all the histopathological evaluation embodied in this study. JLF provided clinical specimens and patient data. PGW conceptualized the study, provided the infrastructure and funding support, supervised the work and data analysis and edited the manuscript. All the authors read and approved the manuscript.

## Acknowledgements

The financial support of this work from Mount Sinai Foundation of Toronto, Da Vinci Gala Fundraiser, Alex and Simona Shnaider Chair in Thyroid Cancer, The Temmy Latner/Dynacare Foundation, and the Mount Sinai Hospital Department of Medicine Research Fund is gratefully acknowledged. We thank Dr. Ajay Matta for help in carrying out the statistical analysis.

## Pre-publication history

The pre-publication history for this paper can be accessed here:

http://www.biomedcentral.com/1471-2407/10/331/prepub
